# Vitamin K2-Dependent GGCX and MGP Are Required for Homeostatic Calcium Regulation of Sperm Maturation

**DOI:** 10.1016/j.isci.2019.03.030

**Published:** 2019-03-29

**Authors:** He Ma, Bao Li Zhang, Bao Ying Liu, Shuo Shi, Da Yuan Gao, Tian Cheng Zhang, Hui Juan Shi, Zhen Li, Winnie Waichi Shum

**Affiliations:** 1 Department of Histology and Embryology, Fourth Military Medical University, Xi'an 710032, China; 2 School of Life Science and Technology, ShanghaiTech University, Shanghai 201210, China; 3 CAS Center for Excellence in Molecular Cell Science, Shanhai Institute of Biochemistry and Cell Biology, Chinese Academy of Science, Shanghai 200031, China; 4 University of Chinese Academy of Sciences, Beijing 100049, China; 5 Shanghai Institute for Advanced Immunochemical Studies, ShanghaiTech University, Shanghai 201210, China; 6 Key Lab of Reproduction Regulation of NPFPC-Shanghai Institute of Planned Parenthood Research, Reproduction and Development Institution, Fudan University, Shanghai 200032, China

**Keywords:** Cell Biology, Developmental Biology, Specialized Functions of Cells

## Abstract

A low-calcium microenvironment is essential for spermatozoa to mature in the epididymis; however, it remains unclear how dysregulation of epididymal luminal calcium is associated with male infertility. Using a warfarin-induced vitamin K2 deficiency rat model, we found that vitamin-K-dependent γ-glutamyl carboxylase (GGCX) and matrix Gla protein (MGP) were essential in extracellular calcium signaling of the intercellular communication required for epididymal sperm maturation. We found that GGCX and MGP co-localized in the vesicular structures of epididymal cells and spermatozoa. Calcium-regulated MGP binds to proteins in a biphasic manner; sub-millimolar calcium enhances, whereas excessive calcium inhibits, the binding. Bioinformatic analysis of the calcium-dependent MGP-bound proteome revealed that vesicle-mediated transport and membrane trafficking underlie the intercellular communication networks. We also identified an SNP mutation, rs699664, in the *GGCX* gene of infertile men with asthenozoospermia. Overall, we revealed that the GGCX-MGP system is integrated with the intercellular calcium signaling to promote sperm maturation.

## Highlights

• Epididymal sperm maturation requires VK2-dependent GGCX-mediated MGP carboxylation
• A GGCX SNP mutation is found in infertile men suffering from asthenozoospermia
• Carboxylated-MGP regulates intercellular calcium signaling in the epididymal lumen
• Calcium-regulated MGP binds to proteins in a biphasic-manner and favors low levels

## Graphical Abstract

**Figure:**
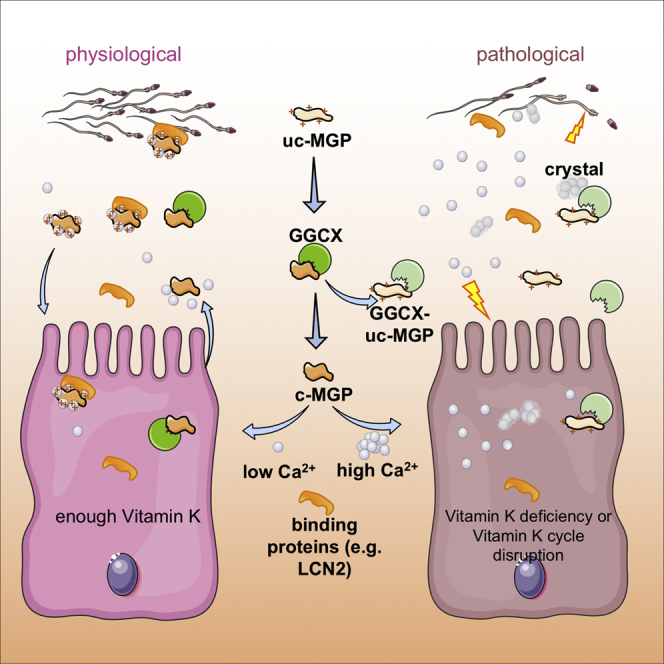

## Introduction

Proper intercellular communication is essential for multicellular organisms to function and survive normally, and aberrant exchange of information may result in pathological conditions and diseases. Extracellular vesicles (EVs; also known as exosomes and microvesicles) are the information carriers for cell-to-cell communication in most cell types (Schneider and Simons, 2013, Tkach and Thery, 2016). One cell type that heavily relies on intercellular communication is the spermatozoon, which is generated in the testis and whose transcription is halted, for the cell cycle is arrested until fertilization (Braun, 1998). Spermatozoa have to exchange numerous cellular components by interacting with the epithelial cells that nurture and protect them while they transit through and mature in the epididymis (Dacheux and Dacheux, 2014, Hermo and R.B, 2002, Robaire and Hinton, 2015). The cell-cell cross talk among spermatozoa and the various types of epididymal cell takes place in the luminal microenvironment, in which a unique composition of epididymal fluid is formed and regulated for special needs of these cells, such as metabolic demands (Cornwall, 2009, Robaire and Hinton, 2015, Turner, 2002, Zhou et al., 2018). The epididymal epithelium, at the front line in contact with spermatozoa, is responsible for the formation of the epididymal luminal microenvironment and conveying the epigenetic and environmental factors to the spermatozoa and thus plays a critical role in the regulation of male fertility as well as the health of offspring (Chen et al., 2016a, Chen et al., 2016b, Conine et al., 2018, Hansen et al., 2010, Latchoumycandane et al., 2002, Sharma et al., 2016, Sharma et al., 2018, Smythies et al., 2014, Sun et al., 2018). However, the physiological roles of the constituents of the epididymal luminal microenvironment, the medium for the communication between spermatozoa and epididymal cells, remain largely unknown.

One feature of the epididymal luminal microenvironment is a low level of calcium (Cornwall, 2009, Levine and Marsh, 1971, Turner, 2002). An aberrantly high Ca^2+^ level in the lumen can result in defective spermatozoa and impaired fertility, without affecting spermatogenesis (Luconi et al., 1996, Schuh et al., 2004). The epithelial calcium channel TRPV6 has been reported to be important for epididymal luminal calcium homeostasis (Weissgerber et al., 2011). However, we have found that TRPV6 co-localizes with the calcium-activated anion channel TMEM16A in some epididymosomes of the very proximal and the very distal epididymis. This co-localization is absent from the caput and proximal corpus epididymidis where supposedly most of the calcium is reabsorbed (Gao da et al., 2016). This suggests the presence of another calcium reabsorption mechanism in addition to the TRPV6-mediated pathway. It is known that vitamin K (VK) is a key modulator of extracellular calcium homeostasis in maintaining bone health and preventing ectopic calcification in blood vessels and other systems (Schurgers et al., 2013, Wallin et al., 2008, Weber, 2001). We therefore examined whether the vitamin-K-dependent (VKD) calcium regulation mechanism also functions in the regions of epididymis where the TRPV6-TMEM16A coupled complex is absent, and, if so, how VKD calcium regulation is involved in the cell-cell communications that take place in the epididymal luminal microenvironment.

The epididymal epithelium actively secretes and absorbs epididymosomes, the EVs in the epididymal luminal microenvironment (Chen et al., 2016a, Conine et al., 2018, Sharma et al., 2016, Sharma et al., 2018, Sullivan et al., 2005). Interestingly, EVs are also known as matrix-calcifying vesicles (Lotvall et al., 2014), an annotation suggesting that EVs and extracellular calcium homeostasis are functionally correlated. EVs are released in response to environmental stress by factors promoting calcification (Kapustin et al., 2011, Kapustin et al., 2015, Reynolds et al., 2004). In the vasculature, released EVs are loaded with MGP, a VK2-dependent calcification-inhibiting protein that requires the carboxylation by GGCX to prevent extracellular calcification (Reynolds et al., 2004). We speculate that GGCX and MGP are important for homeostatic calcium regulation in the epididymis, a mechanism that has not previously been studied.

To sustain the carboxylation activity, GGCX relies on the VK cycle, in which VK is reduced by vitamin K epoxide reductase (VKOR) and continues the carboxylation reaction (Rishavy and Berkner, 2012, Schurgers et al., 2013, Stafford, 2005, Tie and Stafford, 2016). Warfarin is the specific inhibitor of the VKOR complex subunit 1 (VKORC1) able to interfere with the VK cycle (Hirsh et al., 2001, Piatkov et al., 2010). We therefore used the warfarin-induced VK2 deficiency model of adult rats to examine if GGCX and MGP participate in homeostatic calcium regulation in the epididymis.

Overall, our data reveal a role of VK2-dependent GGCX-MGP pathway in extracellular calcium-dependent vesicle-mediated cell-to-cell communication during sperm maturation. In extending the biological relevance of VKD-MGP signaling from rats to humans, we also identified an SNP mutation of *GGCX* rs699664, which has been reported to cause a change from 325Arg to Gln, in association with idiopathic asthenozoospermia in infertile patients. Our study suggests that mutations of GGCX or dysfunction of the GGCX-MGP system in calcium-regulated epididymal sperm maturation is the pathological cause in some infertile men.

## Results

### Dynamic Cellular Localization of GGCX and MGP in the Rat Epididymis

**Figure 1:**
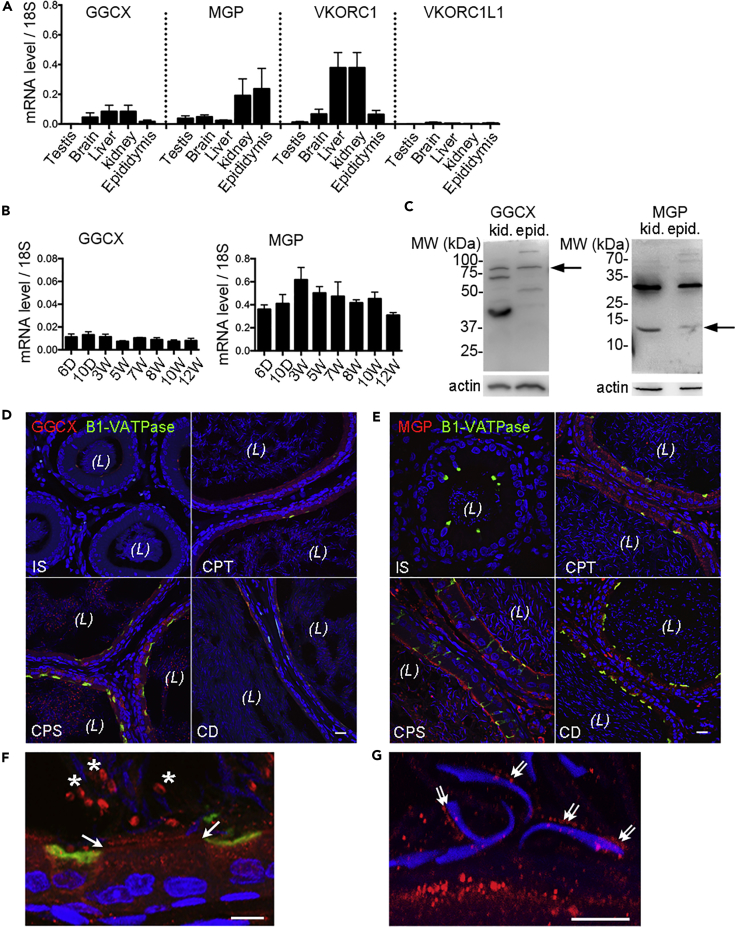
The Expression Patterns of Vitamin K Cycle-Associated Proteins in Rats (A) qPCR of GGCX, MGP, VKORC1, and VKORC1L1 mRNA in different tissues of adult WT rats. (B) GGCX and MGP mRNA in the key postnatal stages (days 6–10 and weeks 3–12) of the rat epididymis. Results are means (±SD) of three rats in panels A and B. (C) Immunoblot of GGCX and MGP proteins in the tissue lysates of the epididymis and kidney from WT rats. Arrows indicate the bands of expected size of GGCX (∼88 kDa) and MGP (∼12 kDa). Actin serves as the loading control. Note the additional band detected at ∼32 kDa by MGP antibody larger than its expected size, a characteristic of MGP-mediated calcium-dependent complex aggregation as described in this study. (D and E) Immunolabeling of GGCX (D, red) and MGP (E, red) proteins in cryosections of the WT adult rat epididymis. CD, cauda epididymidis; CPS, corpus; CPT, caput; IS, initial segment. Clear cells are labeled for B1-VATPase in green. Nuclei and spermatozoa heads are labeled with DAPI in blue. (*L*), lumen. Scale bars, 20 μm. (F) Higher magnification showing GGCX immunolabeling in the granular organelles of principal cells. Arrows: apical stereocilia or principal cells; asterisks: the cytoplasmic droplets of spermatozoa. (G) Higher magnification showing the MGP-labeled vesicular structures (double arrows) at the head of spermatozoa near the apical domain of principal cells. Scale bars,10 μm in F and G.

As a first step to determine the expression level of the VK-cycle-associated proteins in the epididymis, real-time PCR method was used, and results showed that the mRNAs of GGCX, MGP, and VKORC1 were all expressed in the wild-type (WT) rat epididymis, whereas VKORC1L1 was detected at a negligible level (Figure 1A). The mRNA levels of the GGCX substrate MGP were about 10-fold higher in the epididymis than in the testis and other organs. High MGP mRNA expression was also detected in the kidney, an organ sharing many similar physiological properties with the epididymis. GGCX mRNA expression was stable throughout postnatal development of the epididymis, whereas MGP mRNA increased moderately at early ages then decreased slightly toward adulthood (Figure 1B). Western blots of rat epididymis and kidney showed the expected band for GGCX at about ∼88 kDa and for MGP at about ∼12 kDa (Figure 1C, arrows). The bands below the expected size of GGCX might represent its degradation products, as demonstrated in other studies (Tie et al., 2016). Noteworthy, a strong band was detected above the expected size of MGP at ∼32 kDa, which is a common characteristic of immunoblotting for MGP observed in other studies (Lomashvili et al., 2011), and in this study it was attributable to an MGP-mediated calcium-dependent protein complex, described in the subsequent section.

To determine the cellular distribution and subcellular localization of the proteins, we performed confocal imaging and immunofluorescence labeling experiments. The results showed that both GGCX and MGP proteins were absent when the spermatozoa first entered the initial segment, but they appeared strongly in the epithelial cells of the caput and corpus epididymidis, then slightly decreased in the cauda epididymidis of WT males (Figures 1D and 1E). No MGP was detected in basal cells (Figure S1). Higher-magnification images showed that GGCX was mainly located in the intracellular granular organelles of epithelial cells, particularly enriched in the apical stereocilia of principal cells as well as in the cytoplasmic droplets of spermatozoa in the lumen (Figure 1F, arrows and asterisks). Consistent with the notion of a secretory protein on EVs, MGP immunolabeling was accumulated in the vesicular structures of the apical membrane of principal cells and in the luminal contents, especially in the corpus region. The MGP-positive vesicle-like structures were not only enriched in the apical membrane punctae of principal cells but were also aligned in rows on the surface of the sperm head (Figure 1G, double arrows).

**Figure 2:**
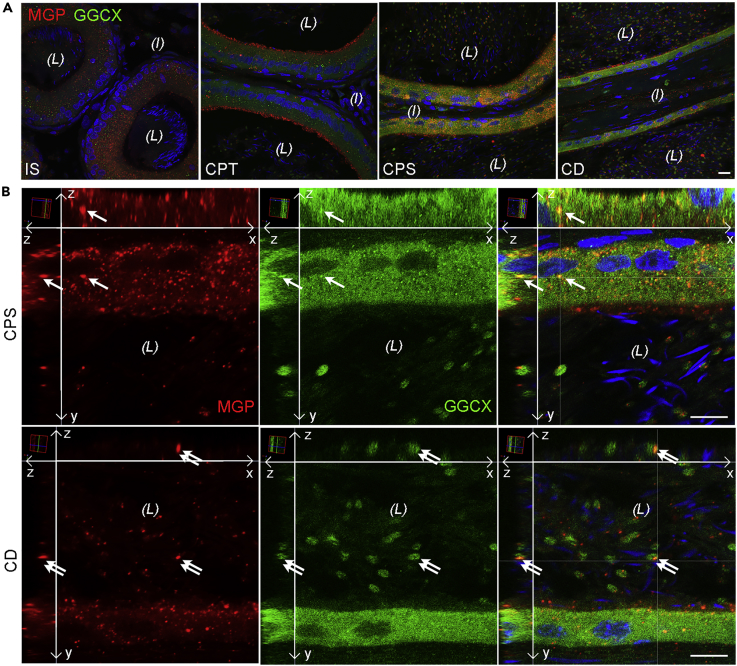
Co-localization of MGP and GGCX Proteins in Vesicular Structures of Epithelial Cells and Spermatozoa in the Epididymis (A) Representative projections of stacks of serial confocal images showing the double-immunofluorescence labeling of MGP (red) and GGCX (green) in the different regions of the rat epididymis. CD, cauda epididymidis; CPS, corpus; CPT, caput; IS, initial segment. *(L)* lumen; *(I)* interstitial tissue. Scale bar, 20 μm. (B) Three-dimensional reconstruction and orthogonal views (xy, xz, yz) of higher magnification of stacks of confocal images demonstrating the co-localization of MGP and GGCX (yellow) in both intracellular and extracellular vesicular structures of epithelial cells (arrows) and cytoplasmic droplets (double arrows) of spermatozoa in the lumen of CPS and CD. Long arrows indicate the xy, xz, and yz dimensions. DAPI (blue) was used to visualize nuclei and sperm heads. (*L*), lumen. Scale bars, 10 μm.

To determine the cellular colocalization of MGP and GGCX, double-immunofluorescence labeling of rat epididymal cryosections were performed (Figure 2). Colocalization patterns of MGP and GGCX were enriched in the corpus and cauda epididymidis, but scarce co-localization was observed in the initial segment and caput regions (Figure 2A). Three-dimensional projections of higher-magnification micrographs showed that the colocalization was found in vesicular structures of the epithelial cells and the cytoplasmic droplets of spermatozoa in the lumen (Figure 2B). These data suggest that *in situ* MGP carboxylation by GGCX occurs not only in epididymal epithelial cells but also in the epididymal luminal microenvironment.

### Defective Epididymal Sperm Function and Male Fertility after Inhibition of GGCX by Warfarin Administration to Rats

**Figure 3:**
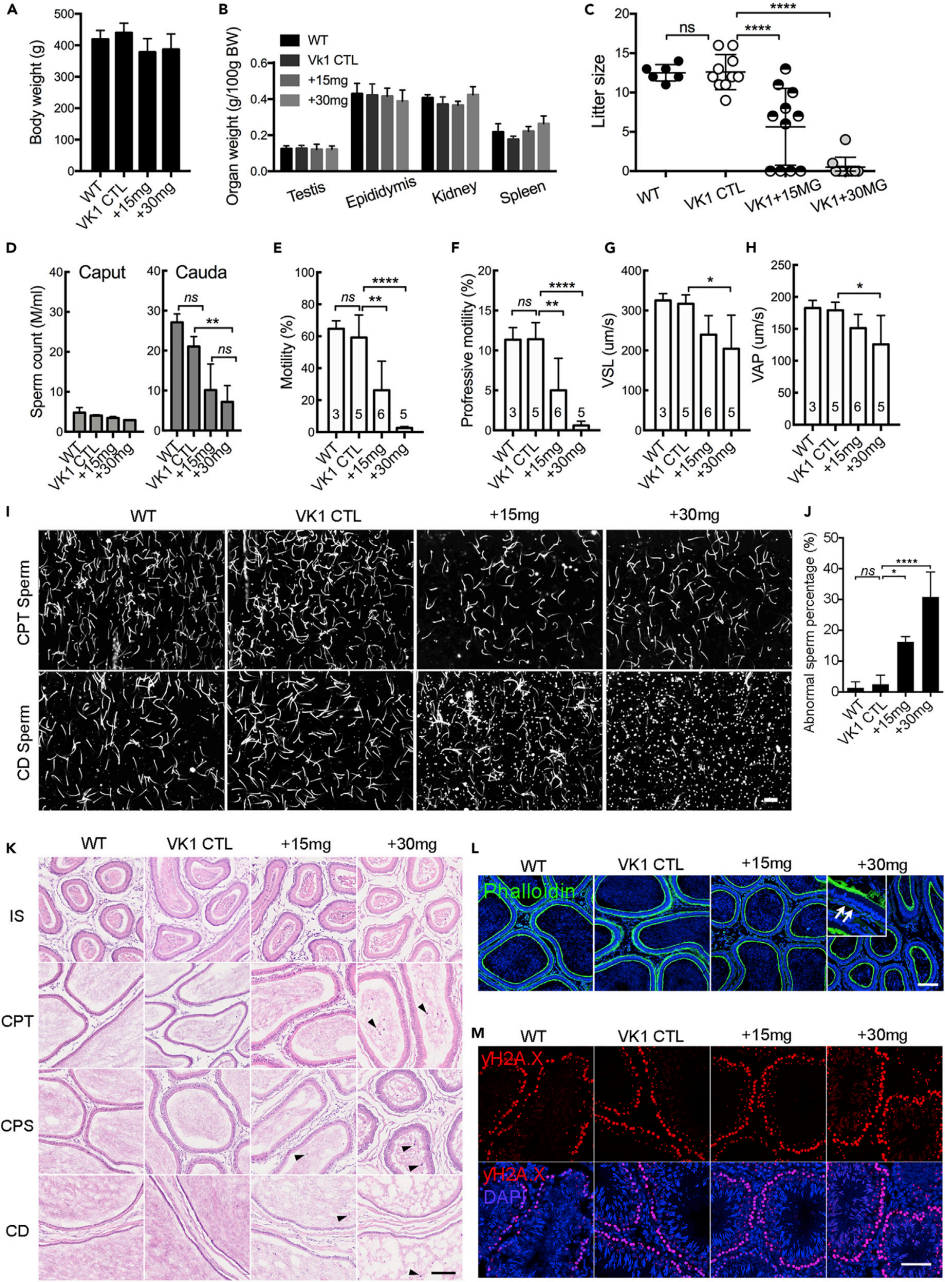
Functional Analysis of Warfarin-Treated Rat Epididymal Sperm and Male Fertility (A–C) Body weights (A), organ-to-body weight ratios for the testes, epididymis, kidneys, and spleens (B) as well as the litter size (C) of rats from +15mg and +30mg warfarin-treated groups versus WT and VK1 supplemented control groups. (D) CASA quantification of the concentration of motile caput and cauda spermatozoa. (E–H) The percentage of motile (E) and progressive (F) sperm and the comparison of VSL (G) and VAP (H) parameters of cauda sperm from warfarin-treated and control rats. (I) Images of caput and cauda sperm during CASA experiments. Note the numerous granular vesicles in the contents of cauda epididymidis of the warfarin-treated rats but not in the controls nor the caput contents. (J) Percentage of defective cauda sperm from warfarin-treated rats versus controls. (K) H&E staining showing gross anatomy of epididymis of each group. Arrowheads indicate the detached epithelial cells in the lumen. (L) Immunofluorescent labeling showing the reduced F-actin in the smooth muscle layer (arrows) in the basal compartment of warfarin-treated epididymis. (M) Immunolabeling of γ-H2AX (red) in testicular sections showing normal spermatogenesis in the testes of each group. Nuclei are labeled in blue with DAPI. Scale bars, 50 μm. Bar graph data herein and in subsequent figures show the means (±SD) of at least three rats or trials; *ns:* no significant difference; *p < 0.05, **p < 0.01, ***p < 0.001, ****p < 0.0001.

To investigate the reproductive function of the VKD proteins GGCX and MGP *in vivo*, we established a warfarin-induced VK2-deficient rat model by using a modified protocol supplemented with VK1 to avoid VK1-deficiency-induced hemorrhage, as described previously (Price et al., 1998). Compared with the WT rats, all rats in the VK1 control (VK1 CTL) and VK1 co-administered with 15 mg (+15 mg) and 30 mg (+30 mg) warfarin showed normal body weight (Figure 3A) and expected organ-to-body ratios for the testes, epididymides, kidneys, and spleens (Figure 3B). To evaluate fertility (Figure 3C), adult male rats in each group were mated with WT female rats. The mean litter size in the +15 mg and +30 mg groups was significantly smaller in a dosage-dependent manner than in control groups, whereas the rats in the VK1 control group had normal litter sizes, as did those of the WT rats. To elucidate the defects underlying the subfertility, the numbers of spermatozoa from the caput and the cauda epididymidis were measured (Figure 3D). The results showed that warfarin treatment reduced the cauda sperm count in a dosage-dependent manner, whereas the caput sperm count showed no significant difference. The assessment of cauda epididymidal spermatozoa by computer-assisted semen analysis (CASA) revealed that the percentage of total and progressively motile spermatozoa, straight line velocity (VSL), and averaged path velocity (VAP) were significantly decreased in the warfarin-treated groups compared with those of the control groups (Figures 3E–3H). Images captured during CASA showed that cauda but not caput spermatozoa were sluggish and frequently aggregated (Figure 3I). Numerous granules were accumulated around spermatozoa in the cauda epididymidal lumen of the warfarin-treated groups, also in a dose-dependent manner. The cauda spermatozoa of +15 mg and +30 mg treated rats displayed a significantly higher percentage of missing heads or a bent head wrapped around the neck when compared with controls (Figure 3J). Gross anatomical analysis of epididymal cryosections showed that there were some detached epithelial cells in the lumen of the caput, corpus, and cauda regions of warfarin-treated rats, whereas they were seldom found in the initial segment (Figure 3K). It was noted that the F-actin cytoskeleton immunofluorescent signals were also significantly reduced in the smooth muscle cells of warfarin-treated rats, whereas no obvious difference was found in the control groups (Figure 3L). Spermatogenesis in the testis was unaffected in any group (Figure 3M).

### Dysregulation of Epididymal Luminal Microenvironment and Homeostatic Calcium Dysregulation in the Epididymis of Warfarin-Treated Rats

**Figure 4:**
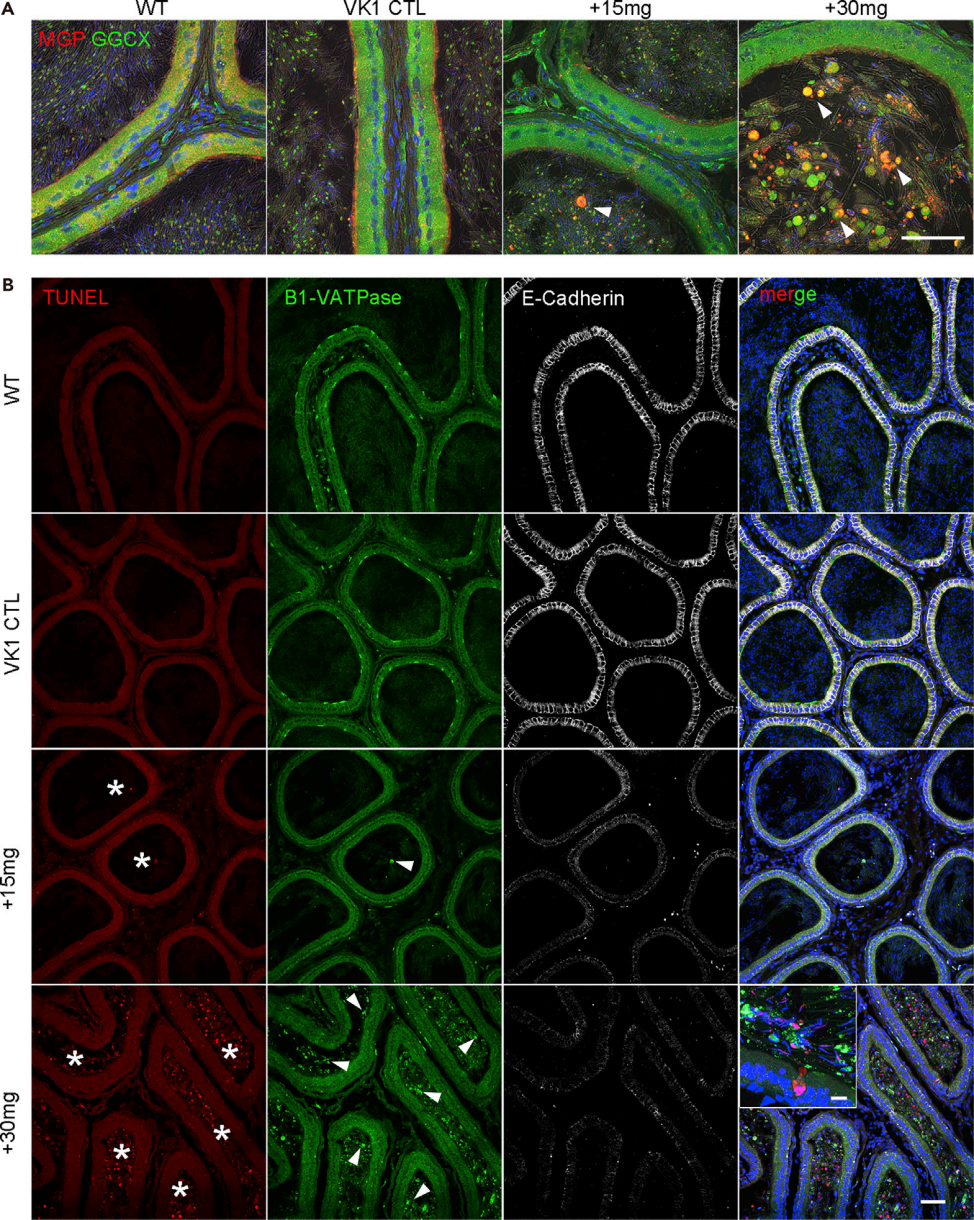
Warfarin-Treated Rats Show Microenvironment Disorders and Apoptotic Epididymal Epithelial Cells (A) Confocal projection and superimposed differential interference contrast images of CPS cryosections double immunolabeling for MGP (red) and GGCX (green) of rats under wild-type, VK1 control, +15 mg, and +30 mg warfarin conditions. Arrowheads indicate the shedding of epithelial cells and larger vesicles into the lumen of warfarin-treated epididymides. (B) Triple-fluorescent labeling for TUNEL (red), clear cell marker B1-VATPase (green) and epithelial cell marker E-Cadherin (white) of epididymal cryosections of the different groups. Note the reduced signals of B1-VATPase and E-cadherin in the epithelium of +15 mg and +30 mg warfarin-treated groups in a dosage-dependent manner. There are numerous TUNEL-positive cells (asterisks) and some B1-VATPase positive cells (arrowheads) in the lumen, particularly in the +30 mg group. Inset: higher magnification image showing the +30 mg treated epididymis. Scale bars, 50 μm in A and B, 10 μm in the inset.

The male subfertility, reduced sperm count, defective sperm motility, and exfoliation of the epididymal epithelium in warfarin-treated rats point to a potential disorder of the epididymal microenvironment in these rats. To evaluate this possibility, we performed MGP and GGCX double-immunofluorescence labeling, as well as TUNEL staining of the epididymis. The intracellular co-localization of MGP and GGCX in the epididymal epithelium was decreased, and there was an accumulation of detached cells and larger granules, especially in the epididymis of +30 mg warfarin-treated rats (Figure 4A). The MGP immunofluorescent signals from clear cells and principal cells in the epithelium were reduced dramatically in the +15 mg and +30 mg groups in a dose-dependent manner, suggesting that warfarin treatment caused serious epididymal epithelial cellular dysfunction. TUNEL analysis showed a few apoptotic cells in the epididymal lumen of the +15 mg group and numerous cells in the +30 mg group (Figure 4B).

**Figure 5:**
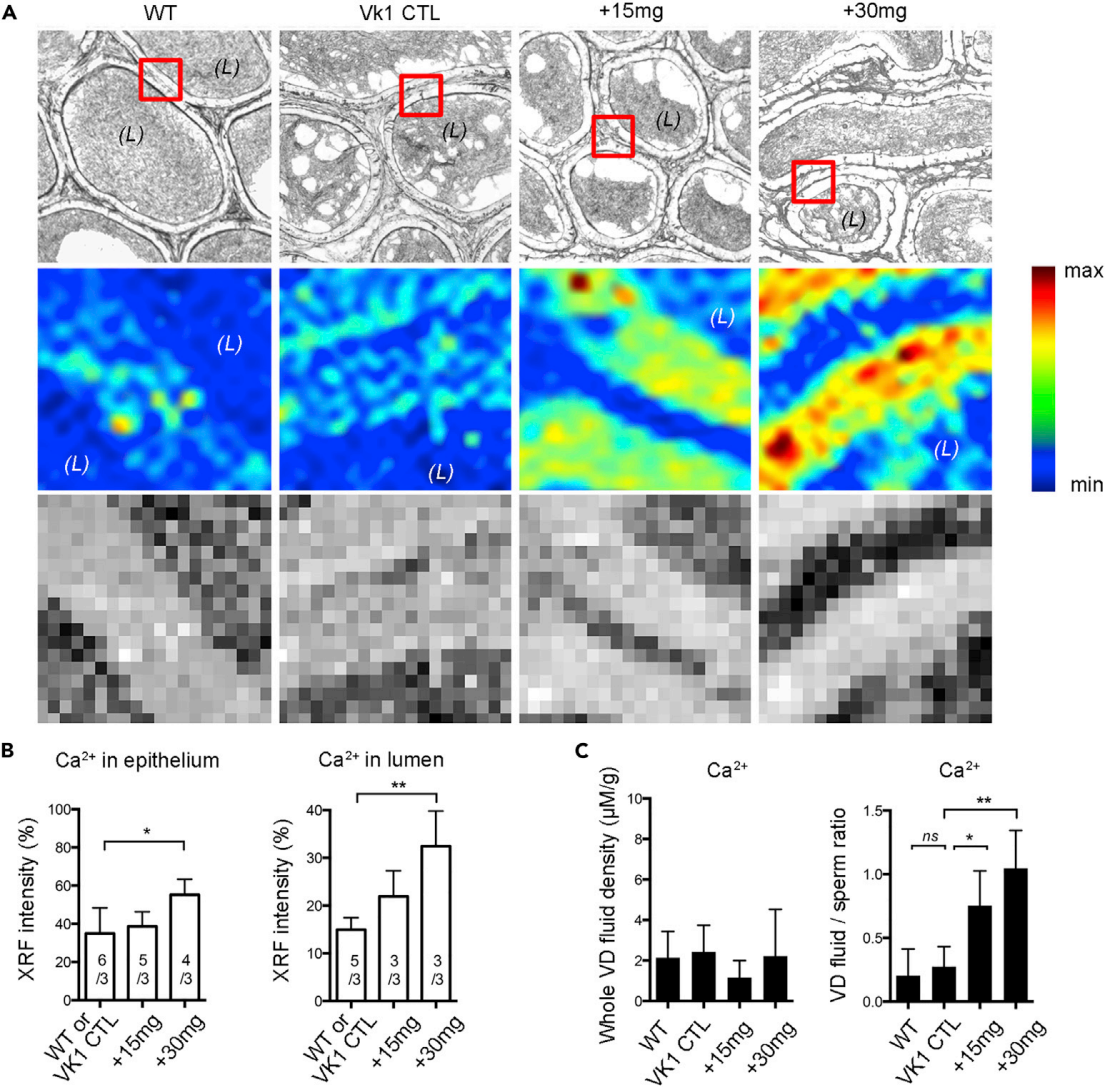
Synchrotron-Based Elemental XRF Imaging and ICP-MS Analysis Revealed Calcium Homeostatic Dysregulation in the Epididymis after Warfarin Administration to Rats (A and B) (A) Light microscopical images arranged in the first row, the boxes indicate the selected regions for XRF analysis in the unstained sections. The second row are XRF elemental maps in the epididymis corpus region of different groups. The elemental maps of Ca^2+^ were acquired using XFM beamline. The third row presents the relative quantitative concentration of Ca^2+^ in the regions and summarized in histograms as in panel B. *(L)*, lumen. (C) ICP-MS analysis of relative Ca^2+^ density in whole VD fluid and the ratio of Ca^2+^ density within sperm-free VD fluid versus spermatozoa.

Unstained epididymal cryosections were analyzed by X-ray fluorescence (XRF) to reveal the relative Ca^2+^ intensity in different groups (Figure 5A). Results were correlated with optical histological images to assess the distribution of elements in the same area of the tissue. The Ca^2+^ concentration tended to be higher in warfarin-treated +15 mg and +30 mg groups than in control rats. Each XRF image was divided into an epithelial portion and a luminal portion for quantification (Figure 5B). The relative Ca^2+^ intensity was about 1.5 and 3times greater, respectively, in the epithelium and luminal area in the +30 mg group than in the control groups. To confirm the Ca^2+^ distribution change in the epididymal lumen after warfarin treatment, epididymal fluid collected from the vas deferens (VD) was analyzed by inductively coupled plasma mass spectrometry (ICP-MS) (Figure 5C). The Ca^2+^ concentration within the VD fluid was not significantly different in any group. However, after eliminating the interference of Ca^2+^ in the spermatozoa, the Ca^2+^ density ratio of VD fluid versus spermatozoa was significantly elevated by about three times in the +15 mg group and four times in the +30 mg group when compared with control groups.

### Decreased Carboxylation Level of MGP in Warfarin-Treated Rats

**Figure 6:**
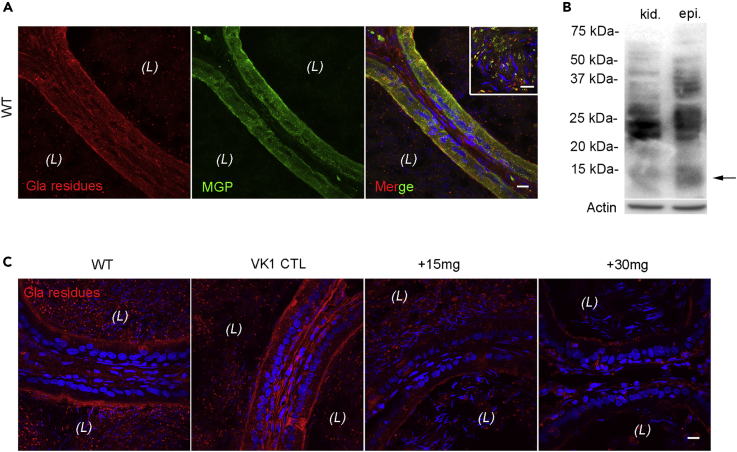
Decreased Protein Carboxylation in the Epididymis of Warfarin-Treated Rats (A) Double immunolabeling with antibodies against Gla-residue-containing proteins (red) and MGP (green) in CPS epididymides showing the similar cellular localization of Gla residues and MGP. Enriched co-localization (yellow) of Gla-residue-containing proteins and MGP in the apical pole of principal cells and luminal vesicular contents (inset). (B) Immunoblot of Gla-residue-containing proteins in rat epididymal and kidney lysates. Arrow indicates the expected size of MGP at ∼12 kDa. Actin: loading control. (C) Immunolabeling of Gla-residue-containing proteins (red) in CPS epididymides of rats from warfarin-treated and control groups. Blue: DAPI labeling for nuclei. (*L*), lumen. Scale bars, 20 μm in A and C, 10 μm in the inset.

To examine the MGP carboxylation status in the epididymis after warfarin treatment, double-immunofluorescence labeling was performed on WT rat epididymal cryosections with an anti-full length MGP antibody and the conformation-specific anti-Gla residue antibody that recognizes proteins containing active γ-carboxyglutamyl (Gla) residues (Figure 6A). Most of the MGP in the WT rat epididymis was recognized by the anti-Gla residue antibody, both in the epithelium and in the lumen. Western blot results confirmed the presence of an anti-Gla residue antibody identified-MGP band at ∼12 kDa (Figure 6B). These results suggest that the extent of binding of the anti-Gla residue antibody reflects the MGP carboxylation status. The immunolabeling of the Gla-residues in the epididymis from the different groups was undertaken to assess the MGP carboxylation level (Figure 6C). The signals from Gla residues were reduced in warfarin-treated epididymides, confirming that warfarin treatment promotes the inactive uncarboxylated form of MGP (ucMGP).

### Identification of Biphasic Calcium-Dependent MGP-Mediated Complex Aggregation and MGP-Binding Targets

**Figure 7:**
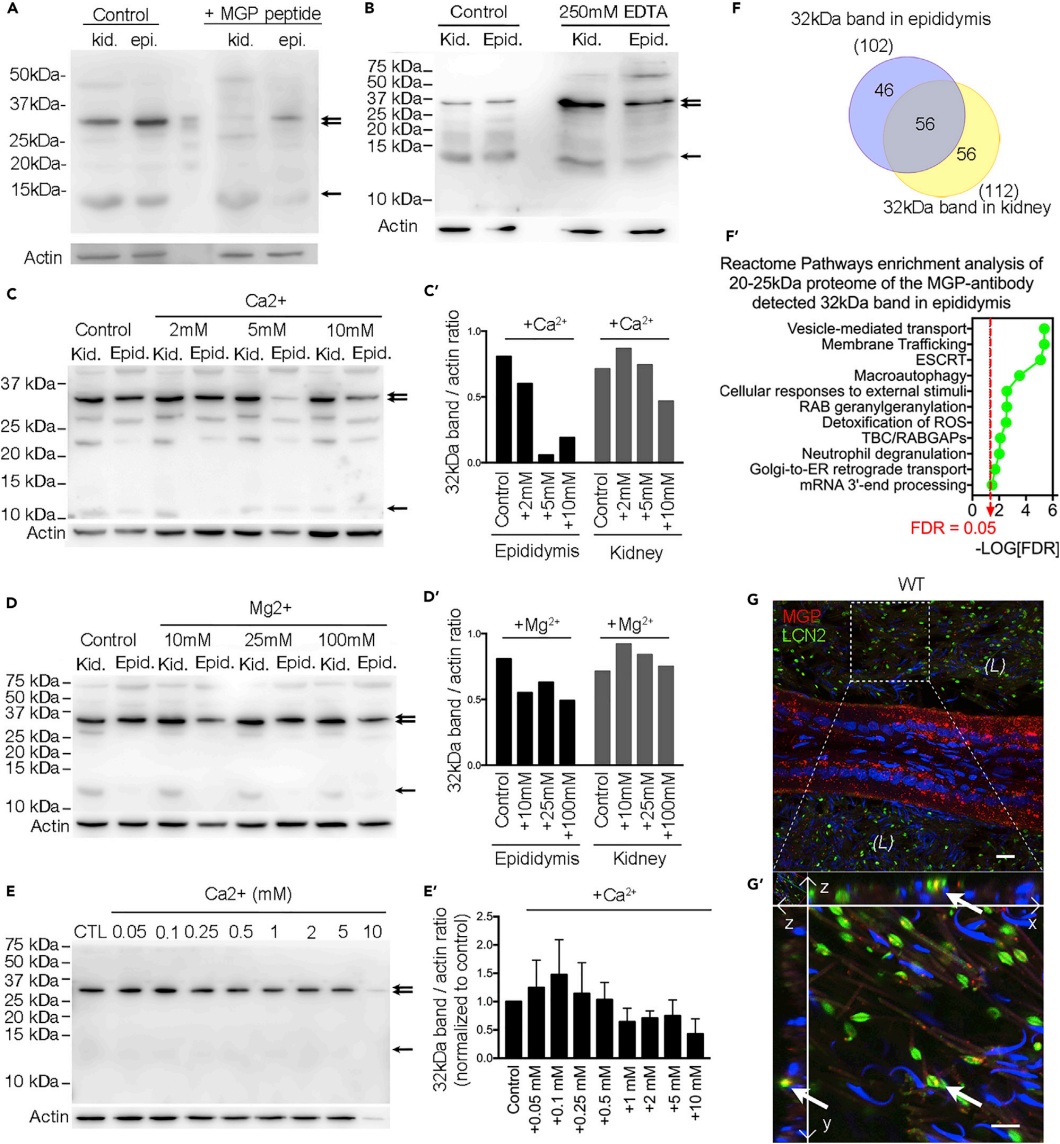
VKD Protein MGP-Mediated Biphasic Calcium-Dependent Complex Aggregation and Binding Target Identification (A) Western blot detection of MGP in total homogenates of kidney and epididymis from WT rats. A band at ∼12 kDa (arrow), corresponding to the molecular weight of MGP, and another band at ∼32 kDa (double arrow), ∼20 kDa larger than MGP, were detected. Both bands were almost abolished by the preincubation with a 10-fold excess of the immunizing peptide of MGP (+MPG peptide) compared with controls. (B) The ∼32 kDa MGP-antibody-detected protein bands (double arrow) were significantly enriched in the presence of 250 mM EDTA compared with controls, whereas the 12 kDa bands remained unchanged (arrow). (C–D′) (C and D) Western blot showing the ∼32 kDa bands in the presence of 2–10 mM Ca^2+^ or 10 to 100 mM Mg^2+^. (C′ and D′) Bar graph showing 32 kDa band/actin intensity ratio in the presence of ions as indicated. (E and E′) (E) Western blot of proteins lysates from DC2 cells in the presence of various calcium concentrations and (E′) the summary of the biphasic effect of externally added calcium on the MGP-detected 32 KDa band intensity. (F and F′) (F) Venn diagram of proteins of molecular size 20–25 kDa detected in the 32 kDa band of the epididymal and kidney lysates analyzed by in-gel digestion and LC-MS/MS. (F′) Reactome pathways enrichments analysis of the candidate proteome of epididymis as in panel F. FDR, false discovery rate. (G and G′) (G) Double immunolabeling of MGP (red) and one of 20 kDa proteins detected in the epididymal ∼32 kDa band, LCN2 (green), in the WT rat corpus epididymidis. (G′) Orthogonal views of high-magnification image showing the predominant colocalization (arrows) of MGP and LCN2 in cytoplasmic droplets of spermatozoa in the lumen. Nuclei and heads of spermatozoa are labeled blue with DAPI. Scale bar, 10 μm.

Western blotting of MGP in the WT rat kidney and epididymis showed two bands at ∼32 and ∼12 kDa. To determine if the ∼32 kDa band was caused by nonspecific binding of anti-MGP antibody, we conducted western blots with MGP antigen peptide (Figure 7A) and found both the 12 and 32 kDa bands were almost diminished, which could mean that the 32 kDa bands contain MGP antigen but are tightly aggregated to other proteins with a size roughly around 20 kDa. Western blot analysis in the presence of extra EDTA, Ca^2+^, and Mg^2+^ (Figures 7B–7D′) showed that the intensity of the ∼32 kDa bands was increased in the presence of 250 mM EDTA (a calcium chelator) in the homogenates, whereas increasing Ca^2+^ from 2 to 10 mM reduced the intensity. The intensity seemed slightly decreased in the presence of Mg^2+^. Increasing the Ca^2+^ concentration from 25 to 100 mM further diminished the amount of the 32 kDa band, as well as most of other proteins including actin, but not the 12 kDa MGP protein (Figure S2). All these results suggest that the MGP aggregate formation with target proteins is dependent on the presence of low calcium concentration and is inhibited by excessive calcium and magnesium. The biphasic calcium dependence of MGP-bound aggregation was confirmed with western blot of protein lysates from immortalized mouse epididymal DC2 cells in the presence of various calcium concentrations (Figures 7E and 7E′).

To identify the target proteins, we analyzed the 32 kDa bands excised from SDS-PAGE gels by in-gel digestion and liquid chromatography-tandem mass spectrometry (LC-MS/MS). The analysis yielded over 1,000 proteins (detected in at least two of five trials of three rounds of proteomic experiments of three animals) in both the 32 kDa bands of epididymis and kidney with molecular sizes ranging from 10–530 kDa (data not shown). Of these proteins, 102 and 112 proteins were identified with molecular size between 20 and 25 kDa in the 32 kDa bands of the epididymis and kidney, respectively (Figure 7F). Bioinformatic analyses using STRING free online software on these proteomes from the epididymis and kidney and their common genes, respectively, were performed. Results showed that sorting complex required for transport (ESCRT) and Rab proteins responsible for vesicle-mediated transport and membrane trafficking, as well as the response to oxidative stimulus, are the key protein-protein interaction networks (Figures 7F’, S3, and S4). As proof of MGP binding with the identified proteins, we picked LCN2 (∼22 kDa) for the verification for its commercially available antibody and high expression level in the epididymis (Figure S5). Double immunofluorescence labeling of MGP and LCN2 revealed the co-localization in the luminal content but not in the epididymal epithelium (Figures 7G and 7G′).

### GGCX Gene Mutations in Infertile Men with Asthenozoospermia

**Figure 8:**
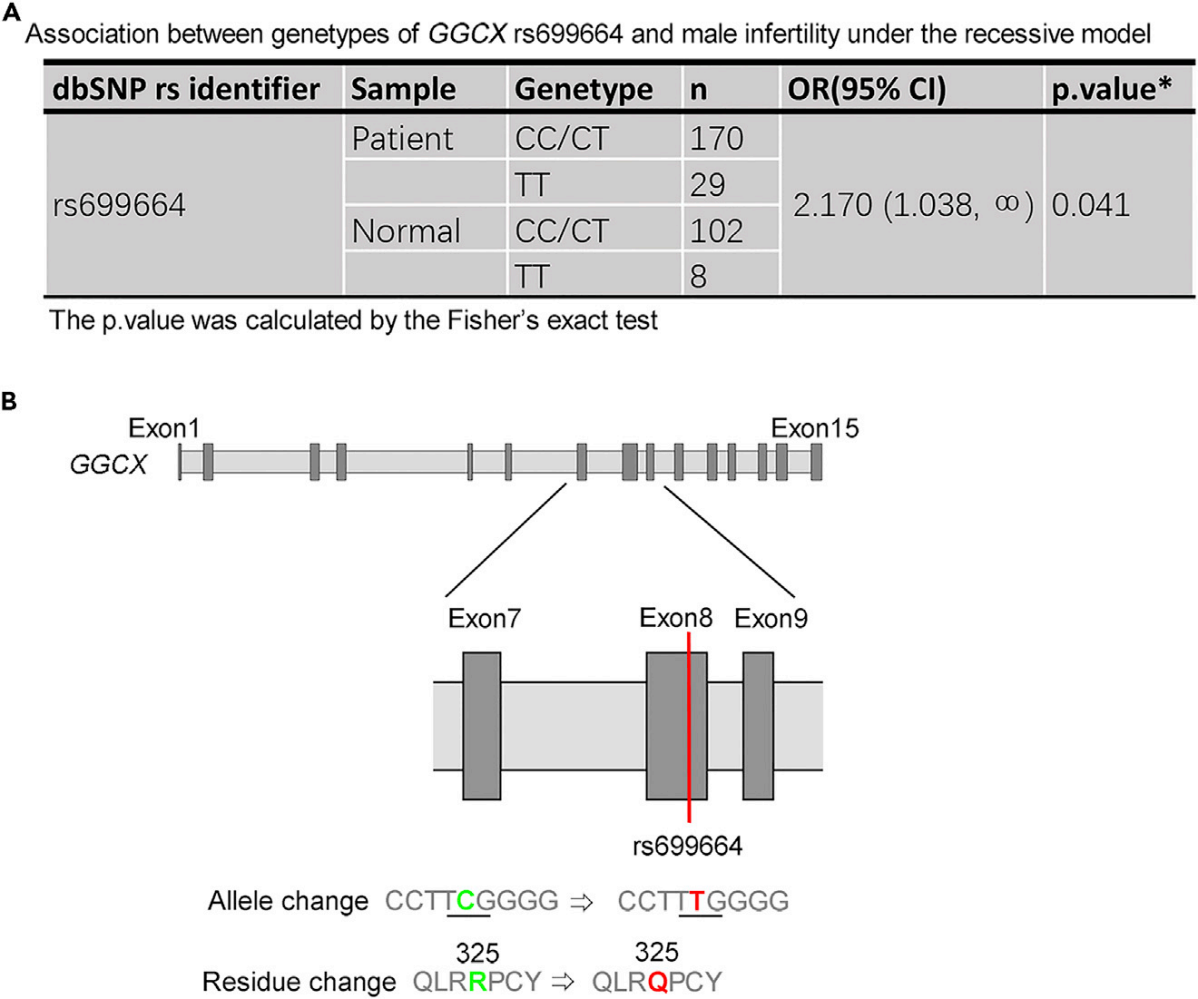
GGCX Gene Mutations in Infertile Male Patients with Asthenozoospermia (A) The association between genotypes of *GGCX* rs699664 and male infertility was assessed in 199 infertile patients with asthenozoospermia and 110 fertile controls under the recessive model. In this the *GGCX* rs699664 is assumed allele T recessive and a one-sided p < 0.05 was considered to be significant. (B) The location of rs699664 is on the eighth exon of GGCX gene with the single nucleotide variation from C (green) to T (red), which results in a residue change from arginine^325^ (R, green) to glutamine^325^ (Q, red).

To check whether mutations of human VKD MGP and GGCX affect male fertility, we screened for potential mutations in the exons of *MGP* and *GGCX* in a cohort of 199 patients with idiopathic asthenozoospermia and 110 fertile controls. The sperm samples collected from fertile healthy donors exhibited normal motility, morphology and sperm counts. We observed a significant association of an SNP mutation rs699664 in *GGCX* with male infertility under the recessive model (Figure 8A). The rs699664 is a non-synonymous T/C SNP in the eighth exon of *GGCX* (Figure 8B), and the allele T is considered regressive (Kinoshita et al., 2007). As reported in the National Center for Biotechnology Information (NCBI) database, this mutation leads to a change from 325Arg to Gln in GGCX, which is in the transmembrane region of GGCX. Our results suggest that the VKD GGCX-MGP system could be a target for clinical screening and potentially for the treatment of idiopathic male infertile cases.

## Discussion

In this study, we demonstrate that the VKD enzyme GGCX and its substrate MGP play a crucial role in the extracellular calcium-dependent vesicle-mediated cell-cell communication involved in the homeostatic regulation of luminal microenvironment and sperm maturation in the epididymis. Both GGCX and MGP proteins are present in vesicular structures in the cytoplasm of epididymal cells and on the surface of spermatozoa in the lumen. This specific cellular localization pattern suggests that GGCX and MGP proteins function in the epididymal lumen microenvironment via the regulation of vesicle-mediated intercellular communication.

It is likely that both GGCX and MGP proteins on the sperm surface and in the cytoplasmic droplets arise from epithelial cells via epididymosomes. Consistently, our proteomic analysis of these biphasic calcium-dependent MGP-binding proteins indicate that vesicle-mediated transport and stress-induced membrane trafficking are the key underlying protein-protein interactions. We speculate that the spermatozoa exert metabolic demands on the epididymal cells as well as provide survival signals to them to sustain a microenvironment that meets the needs of the spermatozoa during their transit through epididymis. We found that optimal extracellular calcium is the mediating cofactor.

By using the warfarin-induced VK2 deficiency rat model, we found that VK cycle disruption resulted in epididymal sperm defects, epithelial exfoliation, and macro-granules accumulation in the epididymal lumen and male infertility. We attribute these phenotypes to the excessive calcium in the epididymis as a result of inactive ucMGP caused by GGCX inhibition after warfarin treatment. The increased ucMGP in the epididymal lumen loses the ability to bind Ca^2+^, which eventually leads to local calcium deposition and promotes calcium phosphate crystal nucleation, as reported from *in vitro* studies (Schurgers et al., 2007, Yagami et al., 1999). Our results therefore showed that VK2 is essential for epididymal sperm maturation and male fertility. Alternatively, they indicated a potential use of warfarin as a male contraceptive by targeting GGCX-MGP.

One unanticipated finding was that, in the MGP western blot assays of epididymal and kidney lysates, we always observed an unknown band at around 32 kDa, a band approximately 20 kDa larger than that of MGP. Our results indicated that this band is a protein complex containing MGP. Moreover, we found that the 32 kDa band was intensified by lowering calcium concentration with the chelation of EDTA and diminished by increasing the concentration of calcium or magnesium in the crude lysates. Although EDTA binds with higher affinity to magnesium than calcium, the higher sensitivity of the 32 kDa MGP band toward calcium suggests that the complex is mainly regulated by calcium rather than by magnesium. The binding of MGP to targeted proteins can also be explained by its special chemical property as well as the unique microenvironment in the epididymis. The inactive form (ucMGP) is a protein with an isoelectric point of 8.8, so that at the acidic pH of ∼6.8 in the epididymal lumen (Breton et al., 1996, Levine and Marsh, 1971, Shum et al., 2009), the ucMGP protein carries five positive charges; the active cMGP would indeed be charge neutral after the addition of five negatively charged gamma-carboxyl groups by GGCX. Thus, cMPG is likely water insoluble, whereas ucMGP is soluble. However, cMGP (carboxylated MGP) has a much stronger binding affinity for divalent calcium ions than ucMGP, and five calcium ions can interact with each MGP molecule (Hackeng et al., 2001, Huang et al., 2003). After calcium binding, cMGP carries ten positive charges, which dramatically increases its water solubility. We speculate that this highly charged cMGP can bind to the negatively charged moieties in the extracellular matrix, including phosphate-containing macromolecules and phosphatidylserine-containing phospholipid membranes, which are present in EVs (Skotland et al., 2018). In another scenario, cMGP could also bind to the inorganic calcium compound hydroxyapatite (Hackeng et al., 2001, Huang et al., 2003), whereas magnesium can interfere with the affinity of cMGP for calcium (Roy and Nishimoto, 2002). It is likely that the high-calcium condition triggers the formation of hydroxyapatite-like compounds that competitively bind to MGP with the organic proteins and subsequently lead to pathological conditions (Tan et al., 2012).

Consistently, our result of titrating calcium concentrations versus the density of the 32 kDa MGP band in cultured DC2 cell lysates (see Figure 7E) revealed that sub-millimolar levels of calcium favor MGP-protein complex formation, which would occur in the sub-millimolar calcium environment of epididymal fluid (Levine and Marsh, 1971, Turner, 2002, Weissgerber et al., 2011). Moreover, when the calcium but not magnesium concentration was increased to as high as 25 to 100 mM, proteins in the lysates could not be electrophoresed normally in the PAGE gel, but the intensity of the ∼12 kDa band was indeed increased compared with actin and the 32 kDa bands, which is believed to be due to the increased solubility of cMGP with excessive calcium in the protein extracts. This also suggests that most MGP is in the active form under physiological conditions of the epididymis and kidney. The diminished 32 kDa bands under high calcium concentration also suggest that the MGP-complex is not susceptible to the reducing conditions of western blot analysis but to the availability of the divalent ions. Taken together, our results show that MGP-mediated protein-complex aggregation is dependent on sub-millimolar calcium levels, whereas excessive calcium levels inhibit aggregation. We propose that, under the physiological low calcium microenvironment of the epididymal lumen, MGP-meditated calcium-dependent macromolecules transport is essential for sperm maturation and plays a critical role in epididymosome-mediated transgenerational information transfer from father to offspring.

To extend our rat study on VKD proteins to human reproductive health, we identified a significant difference in the *GGCX* SNP mutation rs699664 in the asthenozoospermic samples compared with controls, but we found no differences in MGP. It is known that the *GGCX* rs699664 SNP mutation results in the amino acid change from 325Arg to Gln in GGCX (De Vilder et al., 2017) and is associated with bone density in elderly women (Kinoshita et al., 2007, Rieder et al., 2007). Asthenozoospermia is considered a common cause of male infertility characterized by a reduction in both sperm motility and normal sperm morphology (Sanchez-Alvarez et al., 2012). Although the causes of asthenozoospermia are not yet fully understood, an abnormal redox state could be involved in its pathology (Balercia et al., 2004, Sun et al., 2014). Biochemically, the VK cycle is a redox cycle (Ivanova et al., 2018, Stafford, 2005) and it may regulate not only the homeostasis of calcium but also the redox status of cells and tissues.

Previous investigations indicate that MGP's carboxylation status does not affect its secretion into the extracellular environment (Price et al., 1994, Wajih et al., 2004). Consistently, not all MGP in the epididymal lumen is carboxylated. The co-localization of GGCX and MGP suggests that both proteins are secreted in vesicular forms and that uncarboxylated MGP can be activated by GGCX carboxylation *in situ* in the lumen. Therefore, both GGCX carboxylation and the prevention of MGP mineralization may be regulated by the epididymal luminal microenvironment. We speculate that dysregulation of such luminal GGCX-MGP system is the underlying cause of sperm maturation defects from warfarin administration and from the *GGCX* rs699664 mutation. Since the amino acid in position 325 of GGCX is near the MGP's binding site, which is located outside the ER lumen (De Vilder et al., 2017, Tie and Stafford, 2016), it is believed to stay outside of the vesicles and encounter ucMGP in the epididymal luminal microenvironment after its release. It is speculated that the substitution of 325Arg/Gln affects the enzymatic activity of GGCX (De Vilder et al., 2017). Since ucMGP is a peptide containing five positive charges and the 325Arg amino acid of GGCX is singly positively charged and, in addition, it has been hypothesized that all the five γ-glutamyl residues of MGP are carboxylated in one round by GGCX in sequential order (Rishavy and Berkner, 2012), the function of 325Arg in GGCX is therefore likely to stabilize the MGP γ-glutamyl residues binding by dynamic charge-charge interaction with GGCX promoting the sequential carboxylation. The mutation of positively charged 325Arg to a neutral 325Gln moiety in the *GGCX* rs699664 SNP mutation might thereby result in an unfavorable condition for MGP binding during carboxylation, leading to unsuccessful MGP carboxylation and eventually male infertility.

In summary, our study demonstrates that the VK2-dependent protein MGP and its carboxylation enzyme GGCX play an essential role in epididymal sperm maturation and male fertility in rats and that the mutation of GGCX is associated with one form of human male infertility. Our study also suggests that the VKD signaling axis is a promising target for the development of diagnostic products and therapies for male infertility. We also showed that the GGCX-MGP system participates in vesicle-mediated transport, presumably between epithelial cells and spermatozoa in the epididymis and that it is subject to the regulation by luminal calcium in a biphasic manner. In view of the versatile function of EVs in many physiological processes and the ubiquitous expression of MGP, our findings also provide insight into the physiology of VKD GGCX-MGP-dependent calcium signaling in other organs, such as kidney.

### Limitations of the Study

Although this study provided insights into the VKD regulation of epididymal sperm maturation, subsequent studies are necessary to elucidate clearly the molecular mechanism of the GGCX-MGP system for sperm maturation in men. Such studies include providing the direct evidence for the exact roles and contributions of the GGCX mutations, defining the calcium-dependent MGP-mediated intercellular interaction networks, determining the physiological roles of biphasic calcium dependence of MGP-protein complexation, as well as identifying the potential transepithelial transportation pathways for these complexes across epididymis.

## Methods

All methods can be found in the accompanying Transparent Methods supplemental file.

## Supplemental Information

Document S1. Transparent Methods and Figures S1–S5
