# Supplementary material for: Vitamin K2-Dependent GGCX and MGP Are Required for Homeostatic Calcium Regulation of Sperm Maturation
Source: iScience. 2019 Mar 29;14:210–25. doi: 10.1016/j.isci.2019.03.030 (PMC6461585; doi:10.1016/j.isci.2019.03.030)
Supplement: Document S1. Transparent Methods and Figures S1–S5 [file mmc1.pdf]

ISCI, Volume *14*

Supplemental Information

Vitamin K2-Dependent GGCX and MGP Are Required for Homeostatic Calcium Regulation of Sperm Maturation

He Ma, Bao Li Zhang, Bao Ying Liu, Shuo Shi, Da Yuan Gao, Tian Cheng Zhang, Hui Juan Shi, Zhen Li, and Winnie Waichi Shum

Transparent Methods

Study approvals for animals and human samples

The studies involving human subjects were approved by the Medical Ethics Review Board of Shanghai Institute of Planned Parenthood Research. All the individuals signed the informed consent forms. Animal care and use strictly followed institutional guidelines and governmental regulations. Experiments were performed exactly as approved by the IACUC at ShanghaiTech University. All rats were maintained in the animal housing facility provided by Shanghai Model Organisms until end-point experiments.

Animal grouping and warfarin administration

Male Sprague-Dawley rats around 6-7 weeks old were purchased from Shanghai SLAC Laboratory Animal Co. Ltd. Rats of around 8 weeks old were divided randomly into four groups: negative control (WT) without any injection; vitamin K1 control (VK1 CTL) only received 1.5 mg/100g body weight of vitamin K1 (Tokyo Chemical Industry); low dosage warfarin-treated group (+15mg) received 15 mg/100g body weight of warfarin (Tokyo Chemical Industry) and 1.5 mg/100g body weight of vitamin K1; high dosage warfarin-treated group (+30mg) received 30 mg/100g body weight warfarin and 1.5 mg/100 g body weight of vitamin K1. Animals were administrated daily to maintained the designed dosage for consecutive 4 weeks before subjected to fertility test for at least 8-12 days on 1:2 ratio with wild type female rats at age about 10 weeks old, followed by other

endpoint experiments as described below. The experimental procedures to create the vitamin K2 deficiency model were performed on the basis of previous studies with some modifications (Price et al., 1998).

Total RNA extraction and qRT-PCR

Total RNA was extracted from various tissues using Trizol reagent (Invitrogen) according to the manufacture introduction. First-strand cDNA was synthesized using qPCR RT Master Mix (Qiagen). Quantitative RT-PCR analysis was set up with the Prism 7000 Sequence Detection System (Applied Biosystems) using SYBR Green PCR Master Mix (Qiagen). Primers of rat GGCX, MGP, VKORC1 and VKORC1L1 were designed as follows: *ggcx:* 5'-GCTGCTCCCGCCTCAGATAAA-3', 5'-TAAGTA GGGTCACGACACTCT-3'; *mgp:* 5'-CAGCCCTGTGCTATGAATC-3' 5'-AAGG TGTTGGCATTTCTCC-3', *vkorc1:* 5'-GTGTCTGTCGCTGGTTCTC-3', 5'-GCA TTGATGGCATAGGTG-3', *vkorc1l1:* 5'-CTCCATCTACGCCTACCAC-3', 5'-AG ACCAAATCCTCGACCC-3'. The thermal cycling conditions comprised an initial denaturation step at 95°C for 30 seconds, followed by 40 cycles of amplification at 95°C for 5 seconds, 60°C for 30 seconds. The relative transcript levels were quantified using the ΔC_t method with RPS18 (Sangon Biotech) as an internal control.

Immunoblotting

Equal amounts of protein samples of kidney and epididymis were subjected to SDS-PAGE and transfer to PVDF membranes. The membranes were incubated with primary antibodies including anti-GGCX (NBP2-16651, Novus Biologicals; 1:1000), anti-MGP (ab192396, Abcam; 1:2000) and anti-γ-carboxyglutamyl (Gla) residues (3570, Sekisui Diagnostics; 1:500) respectively, further treated with peroxidase-conjugated secondary antibody (711-036-152, Jackson ImmunoResearch; 1:2500). The bound antibodies were visualized by an enhanced chemioluminescence kit (ThermoFisher Scientific). The optical densities of the bands were quantified by using Image J software (NIH).

Immunofluorescence, TUNEL and histological analysis

For immunofluorescence, rat tissues were dehydrated in 30% (w/v) sucrose and cryosectioned in 5-10 µm slices. Sections were hydrated in PBS, antigen retrievaled with 1%w/v SDS, blocked with 1% w/v BSA and then incubated with primary antibodies as follows: GGCX (NBP2-16651, Novus Biologicals; 1:100); MGP (ab192396, Abcam; 1:100), LCN2 (ab63929, Abcam; 1:100); Gla residues (3570, Sekisui Diagnostics; 1:50); γ-H2AX (2577S, Cell Signaling Technology; 1:200); B1-V-ATPase (a gift from Dr. Sylvie Breton, Harvard Medical School; 1:800); E-cadherin (610181, BD Pharmingen; 1:1000). Secondary antibody included FITC-conjugated goat-anti-rabbit or donkey-anti-chicken (111-095-144, 703-095-155, Jackson; 1:50), Cy3-conjugated donkey-anti-rabbit/mouse (711-

165-152/715-165-150, Jackson; 1:800) and Cy5-conjugated donkey-anti-mouse (715-175-151, Jackson; 1:100). The double labeling of two rabbit origin primary antibodies was performed as described previously using a TSA amplification kit (10017450; PerkinElmer) (Gao da et al., 2016). The localization of F-actin of the epididymis and spermatozoa was revealed by using FITC-conjugated phalloidin (P5282-1MG, Sigma; 1:200). Click-iT Plus TUNEL Assay (C10618; Invitrogen) was utilized to detect in suit apoptosis, following the manufacture introduction. Photomicrographs were taken with an A1R (Nikon) confocal microscopy. For histological analysis, epididymal cryoprotected sections (5 μm) were stained with a hematoxylin and eosin kit (60524ES60; Yeasen) and then examined using NanoZoomer (Hamamatsu Photonics).

Mating studies and sperm motility analysis

For fertility analysis, adult male rats (12-13 weeks) in WT, VK1 CTL, +15mg and +30mg groups were paired with WT virgin females (1:2) for 7d. The litter size of each cage was counted within 10d after birth. Spermatozoa were harvested by dissecting the cauda or caput epididymidis in 37°C pre-warmed BWW medium, containing 25 mM KCl, 95 mM NaCl, 1.7 mM $CaCl_2$, 1.2 mM $MgSO_4$, 25 mM $NaHCO_3$, 1.2 mM KH_2PO_4, 5 mM glucose, 0.5 mM sodium pyruvate, 11 mM sodium lactate, 4 mg/mL bovine serum albumin, 0.002% (w/v) phenol red (pH 7.4, the osmolality ~320-330 mosmol/kg). The semen parameters were examined

using a computer- assisted semen analysis system (CASA) (HTM-TOX IVOS sperm motility analyzer, Hamilton Thorne Research).

X-ray fluorescence imaging

Epididymis were cryosectioned at 10 μm thickness and fixed on Mylar thin films. Samples were analyzed at the BL15U1 hard X-ray microprobe at the Shanghai Synchrotron Radiation Facility (SSRF). The incident X-ray energy was set to 12 keV. A Si (111) double-crystal was applied to monochromatize the continuous synchrotron X-ray from an undulator X-ray source. The incident X-ray irradiated on the samples at 45°, and a 50 mm^2 silicon-drift detector was arranged at a 90° angle to the incident X-ray. The samples were raster scanned with a step size of 6 μm × 5 μm and dwell time of one second per step. The relative quantitative concentration of Ca element in different groups was measured by calculating pixels using Excel software. Samples were subsequently captured by optical microscope (Eclipse Ti, Nikon) to align with XRF data.

Microperfusion of the rat vas deferens and Inductively coupled plasma mass spectrometry (ICP-MS)

Microperfusion of the rat vas deferens was performed as previously described (Yeung et al., 2004). Briefly, adult male rats were anesthetized with sodium pentobarbitone by intraperitoneal injection. The very distal cauda epididymidis was isolated together with the proximal vas deferens and cannulated with

polyethylene tubing with a tip diameter of ~300–400 μm, and perfused simultaneously at a rate of ~100 μl/min with air by an infusion pump (Genie Touch, Kent Scientific). The collected sperm-fluid samples were first weighted before diluted 10 times (weight/volume) with 10 mM Tris-HCl (pH 7.4) subsequently centrifuged at 6000 × g, and the supernatant fluid was transferred to a new Eppendorf tube. An equal volume to the supernatant of 10 mM Tris-HCl (pH 7.4) with 1% (v/v) Igepal and 1% (v/v) Triton X-100 was added to, respectively, the supernatant and pellet for solubilization. All the samples were digested through repeated freezing and thawing, and finally diluted to 1 ml with 2% (v/v) nitric acid (MOS grade), stored at 4°C for ICP-MS. The quadrupole-based NexION 2000 S ICP Mass Spectrometer equipped with a TYPE C ST3 Nebulizer and a quartz cyclonic spray chamber (PerkinElmer) was used throughout the experiment. Before analysis, the ICP-MS was tuned by using a 10 mg/L multi-element standard solution. Samples were injected with an autosampler, and ICP-MS was operated in kinetic energy discrimination mode using ultra-high purity ammonia gas with a flow rate of 4.5 ml/min for all metals. ICP-MS operational parameters included: RF power, 1600 W; plasma gas flow, 18 l/min; auxiliary gas flow, 1.20 l/min; and nebulizer gas flow: 0.96 l/min.

In-gel digestion and LC-MS/MS analysis

The in-gel digestion and LC-MS/MS workflow has been described previously (Shevchenko et al., 2006). Briefly, after Coomassie blue staining, the SDS-PAGE

gels containing protein bands of interest were carefully excised, cut into 1 mm×1 mm pieces, and transferred into a microcentrifuge tube. They were destained with 50% (w/v) ammonium bicarbonate/acetonitrile (1:1), dehydrated by the addition of neat acetonitrile, and dried in a vacuum concentrator. Protein disulfide bonds were reduced by 10 mM dithiothreitol at 56 °C for 1 hour and subsequently alkylated with 55 mM iodoacetamide at room temperature for 45 min in darkness. The dry gel pieces were saturated with trypsin buffer followed by overnight incubation at 37°C. The resulting peptide digestion products were extracted in 5% (v/v) formic acid for 15 min in a 37°C shaker. The obtained samples were then subjected to LC-MS/MS analysis with standard protocol for proteomic analysis on an Easy-nLC 1200 system coupled in-line with a Thermo Orbitrap Fusion tribrid mass spectrometer (Thermo Scientific).

Human samples

199 patients with idiopathic asthenozoospermia were recruited from Shanghai Jiai Genetics & IVF Institute. The samples of 110 fertile male controls who had fathered at least one child were obtained in the Shanghai Human Sperm Bank. The genomic DNA was extracted from peripheral blood for SNP screening. All of the exons of the GGCX and MGP gene were amplified by polymerase chain reaction (PCR) using LightCycler 480II (Roche Diagnostics). Then the Illumina HiSeq X10 platform (Illumina) was used to detect SNPs.

Statistical analyses

Data are presented as means ± SD and *P*-values of < 0.05 or below were considered significant. Student's *t*-test was performed to compare the differences between two groups, and multiple comparisons using ANOVA. SNP analysis used association function of SNPassoc package in R version 3.2.4. A one-sided *P*<0.05 was considered to be significant.

Supplemental Figure Titles and Legends

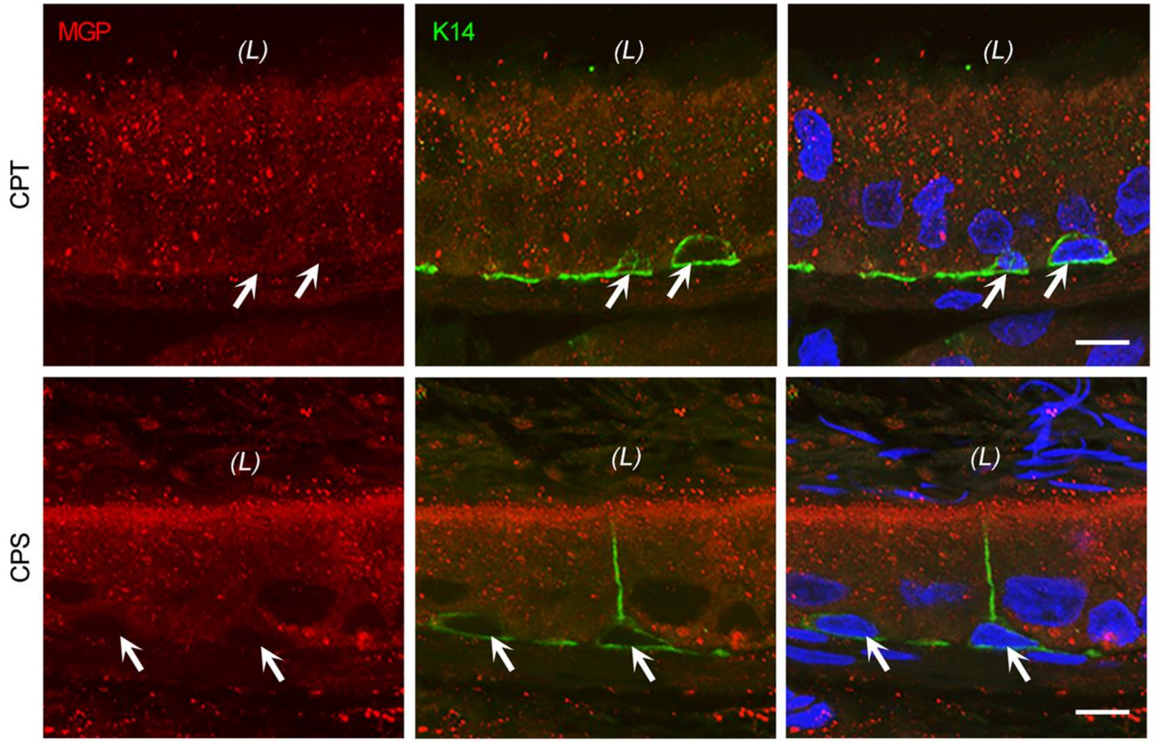

Figure S1. Subcellular localization of MGP in the epididymal epithelial cells, Related to Figure 1. Double-immunofluorescence labelling of MGP (red) and basal cell marker, Keratin-14 (green) in the caput and corpus regions of the rat epididymis. Arrows indicated the basal cells. Nuclei and spermatozoa heads are labeled with DAPI in blue. CPT: caput; CPS: corpus. Scale bars:10 µm.

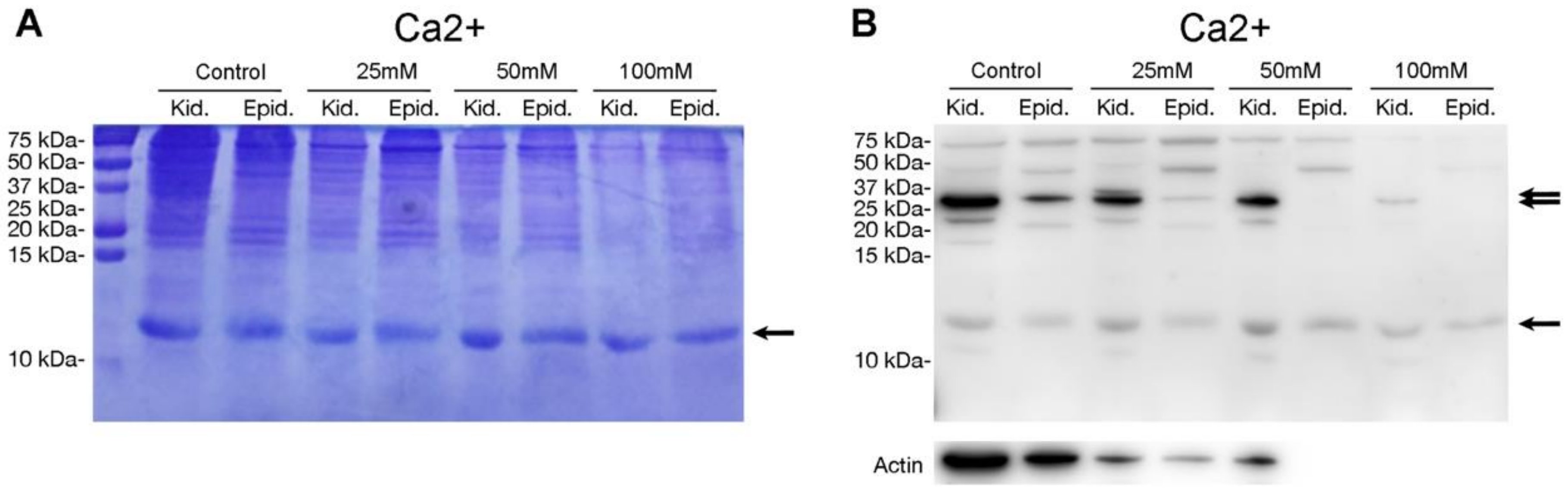

Figure S2. Electrophoresis behaviour of MGP and MGP-bound proteins in the presence of high calcium concentration, Related to Figure 7. (A) Coomassie blue stained SDS-PAGE gel after transfer still showing intensify bands at ~12 kDa corresponding to the molecular weight of MGP (arrow). (B) In the presence high concentration Ca^{2+}, the intensity of ~32 kDa MGP-antibody-detected bands (double-arrow) was significantly diminished while the intensity of ~12 kDa bands remains unchanged or even slightly increased versus actin bands or the ~32kDa bands.

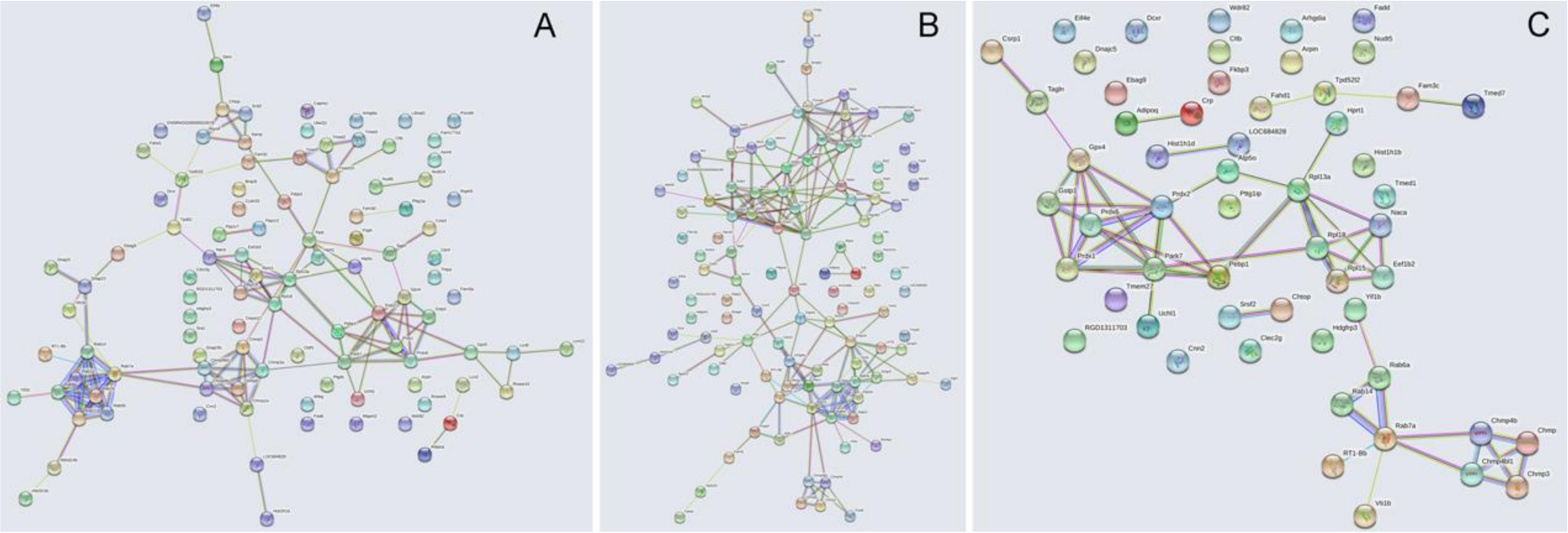

Figure S3. Protein-protein interaction analysis of the 20-25kDa proteome of the 32kDa band detected using antibody raised from full-length MGP in rat epididymis and kidney, Related to Figure 7. (A) The 102 candidate proteins in epididymis. (B) The 112 candidate proteins in kidney. (C) The 56 common candidate proteins detected in both epididymis and kidney. Notably, the proteins involved in Endosomal Sorting Complex Required for Transport (ESCRT) and vesicle-mediated transported pathways, such as Chmp and Rab proteins, are the key common genes found in both epididymis and kidney interaction networks.

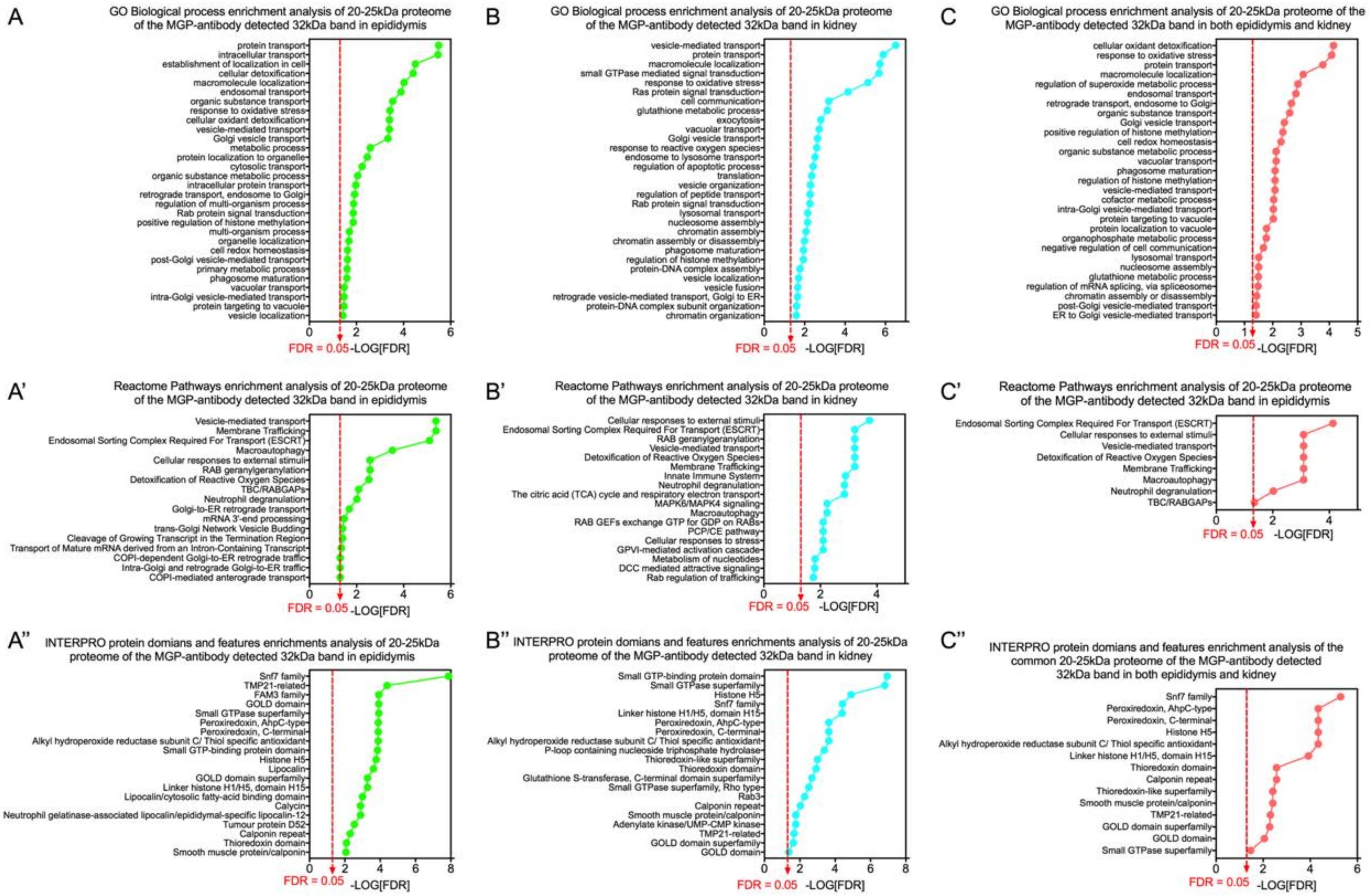
A
GO Biological process enrichment analysis of 20-25kDa proteome of the MGP-antibody detected 32kDa band in epididymis
protein transport
intracellular transport
establishment of localization in cell
cellular detoxification
macromolecule localization
endosomal transport
organic substance transport
response to oxidative stress
cellular oxidant detoxification
vesicle-mediated transport
Golgi vesicle transport
metabolic process
protein localization to organelle
cytosolic transport
organic substance metabolic process
intracellular protein transport
retrograde transport, endosome to Golgi
regulation of multi-organism process
Rab protein signal transduction
positive regulation of histone methylation
multi-organism process
organelle localization
cell redox homeostasis
post-Golgi vesicle-mediated transport
primary metabolic process
phagosome maturation
vacuolar transport
intra-Golgi vesicle-mediated transport
protein targeting to vacuole
vesicle localization
0 2 4 6
FDR = 0.05
-LOG[FDR]
B
GO Biological process enrichment analysis of 20-25kDa proteome of the MGP-antibody detected 32kDa band in kidney
vesicle-mediated transport
protein transport
macromolecule localization
small GTPase mediated signal transduction
response to oxidative stress
Ras protein signal transduction
cell communication
glutathione metabolic process
exocytosis
vacuolar transport
Golgi vesicle transport
response to reactive oxygen species
endosome to lysosome transport
regulation of apoptotic process
translation
vesicle organization
regulation of peptide transport
Rab protein signal transduction
lysosomal transport
nucleosome assembly
chromatin assembly
chromatin assembly or disassembly
phagosome maturation
regulation of histone methylation
protein-DNA complex assembly
vesicle localization
vesicle fusion
retrograde vesicle-mediated transport, Golgi to ER
protein-DNA complex subunit organization
chromatin organization
0 2 4 6
FDR = 0.05
-LOG[FDR]
C
GO Biological process enrichment analysis of 20-25kDa proteome of the MGP-antibody detected 32kDa band in both epididymis and kidney
cellular oxidant detoxification
response to oxidative stress
protein transport
macromolecule localization
regulation of superoxide metabolic process
endosomal transport
retrograde transport, endosome to Golgi
organic substance transport
Golgi vesicle transport
positive regulation of histone methylation
cell redox homeostasis
organic substance metabolic process
vacuolar transport
phagosome maturation
regulation of histone methylation
vesicle-mediated transport
cofactor metabolic process
intra-Golgi vesicle-mediated transport
protein targeting to vacuole
protein localization to vacuole
organophosphate metabolic process
negative regulation of cell communication
lysosomal transport
nucleosome assembly
glutathione metabolic process
regulation of mRNA splicing, via spliceosome
chromatin assembly or disassembly
post-Golgi vesicle-mediated transport
ER to Golgi vesicle-mediated transport
0 1 2 3 4 5
FDR = 0.05
-LOG[FDR]
A'
Reactome Pathways enrichment analysis of 20-25kDa proteome of the MGP-antibody detected 32kDa band in epididymis
Vesicle-mediated transport
Membrane Trafficking
Endosomal Sorting Complex Required For Transport (ESCRT)
Macroautophagy
Cellular responses to external stimuli
RAB geranylgeranylation
Detoxification of Reactive Oxygen Species
TBC/RABGAPs
Neutrophil degranulation
Golgi-to-ER retrograde transport
mRNA 3'-end processing
trans-Golgi Network Vesicle Budding
Cleavage of Growing Transcript in the Termination Region
Transport of Mature mRNA derived from an Intron-Containing Transcript
COPI-dependent Golgi-to-ER retrograde traffic
Intra-Golgi and retrograde Golgi-to-ER traffic
COPI-mediated anterograde transport
0 2 4 6
FDR = 0.05
-LOG[FDR]
B'
Reactome Pathways enrichment analysis of 20-25kDa proteome of the MGP-antibody detected 32kDa band in kidney
Cellular responses to external stimuli
Endosomal Sorting Complex Required For Transport (ESCRT)
RAB geranylgeranylation
Vesicle-mediated transport
Detoxification of Reactive Oxygen Species
Membrane Trafficking
Innate Immune System
Neutrophil degranulation
The citric acid (TCA) cycle and respiratory electron transport
MAPK6/MAPK4 signaling
Macroautophagy
RAB GEFs exchange GTP for GDP on RABs
PCP/CE pathway
Cellular responses to stress
GPVI-mediated activation cascade
Metabolism of nucleotides
DCC mediated attractive signaling
Rab regulation of trafficking
0 2 4
FDR = 0.05
-LOG[FDR]
C'
Reactome Pathways enrichment analysis of 20-25kDa proteome of the MGP-antibody detected 32kDa band in epididymis
Endosomal Sorting Complex Required For Transport (ESCRT)
Cellular responses to external stimuli
Vesicle-mediated transport
Detoxification of Reactive Oxygen Species
Membrane Trafficking
Macroautophagy
Neutrophil degranulation
TBC/RABGAPs
0 2 4
FDR = 0.05
-LOG[FDR]
A''
INTERPRO protein domians and features enrichments analysis of 20-25kDa proteome of the MGP-antibody detected 32kDa band in epididymis
Snf7 family
TMP21-related
FAM3 family
GOLD domain
Small GTPase superfamily
Peroxiredoxin, AhpC-type
Peroxiredoxin, C-terminal
Alkyl hydroperoxide reductase subunit C/ Thiol specific antioxidant
Small GTP-binding protein domain
Histone H5
Lipocalin
GOLD domain superfamily
Linker histone H1/H5, domain H15
Lipocalin/cytosolic fatty-acid binding domain
Calycin
Neutrophil gelatinase-associated lipocalin/epididymal-specific lipocalin-12
Tumour protein D52
Calponin repeat
Thioredoxin domain
Smooth muscle protein/calponin
0 2 4 6 8
FDR = 0.05
-LOG[FDR]
B''
INTERPRO protein domians and features enrichments analysis of 20-25kDa proteome of the MGP-antibody detected 32kDa band in kidney
Small GTP-binding protein domain
Small GTPase superfamily
Histone H5
Snf7 family
Linker histone H1/H5, domain H15
Peroxiredoxin, AhpC-type
Peroxiredoxin, C-terminal
Alkyl hydroperoxide reductase subunit C/ Thiol specific antioxidant
P-loop containing nucleoside triphosphate hydrolase
Thioredoxin-like superfamily
Thioredoxin domain
Glutathione S-transferase, C-terminal domain superfamily
Small GTPase superfamily, Rho type
Rab3
Calponin repeat
Smooth muscle protein/calponin
Adenylate kinase/UMP-CMP kinase
TMP21-related
GOLD domain superfamily
GOLD domain
0 2 4 6 8
FDR = 0.05
-LOG[FDR]
C''
INTERPRO protein domians and features enrichment analysis of the common 20-25kDa proteome of the MGP-antibody detected 32kDa band in both epididymis and kidney
Snf7 family
Peroxiredoxin, AhpC-type
Peroxiredoxin, C-terminal
Histone H5
Alkyl hydroperoxide reductase subunit C/ Thiol specific antioxidant
Linker histone H1/H5, domain H15
Thioredoxin domain
Calponin repeat
Thioredoxin-like superfamily
Smooth muscle protein/calponin
TMP21-related
GOLD domain superfamily
GOLD domain
Small GTPase superfamily
0 2 4 6
FDR = 0.05
-LOG[FDR]

Figure S4. Functional protein association network analysis of the 20-25kDa proteome of the ~32kDa band detected using antibody raised from full-length MGP in the rat epididymis and kidney, Related to Figure 7. Gene Ontology, Reactome Pathways and INTERPRO Protein Domain and Features enrichment analyses using STRING free online software on the 102 candidate proteins in epididymis (A,A',A'') and the 112 candidate proteins in kidney (B,B',B''). (C,C',C'') Gene Ontology enrichment analysis in the 56 common candidate proteins detected in both epididymis and kidney. From the analyses, the significantly enriched gene sets involve the ESCRT, cellular responses to external stimuli and vesicle-mediated transport as revealed by the protein-protein interaction networks.

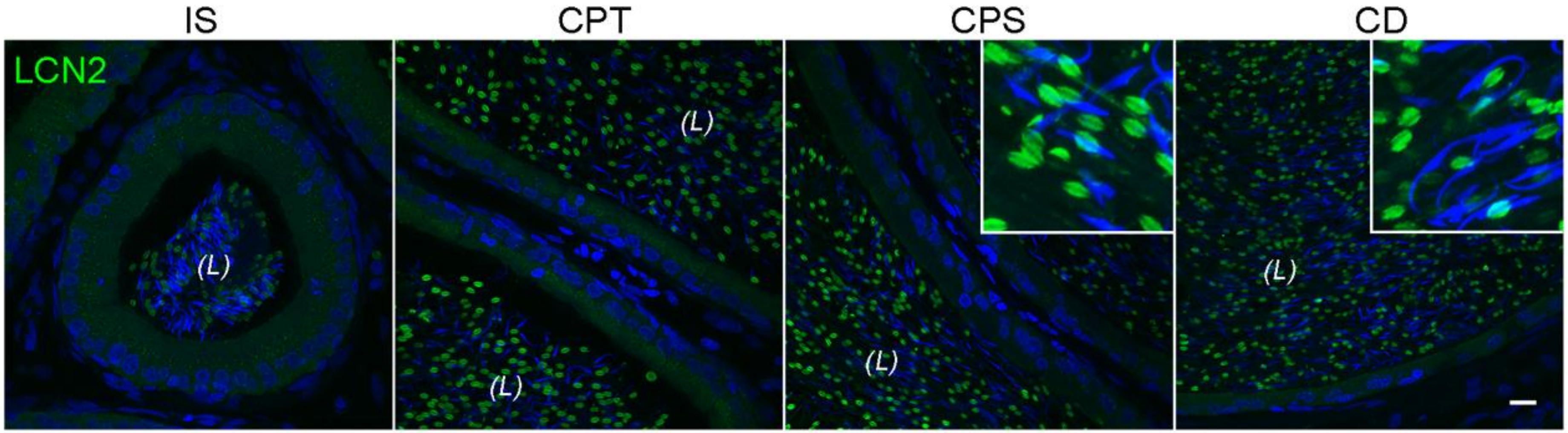

Figure S5. Spatial localization of LCN2 in the rat epididymis, Related to Figure 7. Regional localization of LCN2 (green) in the WT rat epididymis. Nuclei and heads of spermatozoa are labeled blue with DAPI. Scale bars: 20 µm.
